# Clinical applications of machine learning in predicting 3D shapes of the human body: a systematic review

**DOI:** 10.1186/s12859-022-04979-2

**Published:** 2022-10-17

**Authors:** Joyce Zhanzi Wang, Jonathon Lillia, Ashnil Kumar, Paula Bray, Jinman Kim, Joshua Burns, Tegan L. Cheng

**Affiliations:** 1 School of Health Sciences, Faculty of Medicine and Health & Children’s Hospital at Westmead, University of Sydney, Sydney, NSW 2006 Australia; 2 EPIC Lab, Kids Research, The Children’s Hospital at Westmead, Locked Bag 4001, Westmead, Sydney, NSW 2145 Australia; 3 School of Biomedical Engineering, Faculty of Engineering, University of Sydney, Sydney, NSW 2006 Australia; 4 School of Computer Science, Faculty of Engineering, University of Sydney, Sydney, NSW 2006 Australia

**Keywords:** 3D body shape prediction, Machine learning, Regression, Neural network, Artificial intelligence, Surgical planning, Decision making

## Abstract

**Background:**

Predicting morphological changes to anatomical structures from 3D shapes such as blood vessels or appearance of the face is a growing interest to clinicians. Machine learning (ML) has had great success driving predictions in 2D, however, methods suitable for 3D shapes are unclear and the use cases unknown.

**Objective and methods:**

This systematic review aims to identify the clinical implementation of 3D shape prediction and ML workflows. Ovid-MEDLINE, Embase, Scopus and Web of Science were searched until 28th March 2022.

**Results:**

13,754 articles were identified, with 12 studies meeting final inclusion criteria. These studies involved prediction of the face, head, aorta, forearm, and breast, with most aiming to visualize shape changes after surgical interventions. ML algorithms identified were regressions (67%), artificial neural networks (25%), and principal component analysis (8%). Meta-analysis was not feasible due to the heterogeneity of the outcomes.

**Conclusion:**

3D shape prediction is a nascent but growing area of research in medicine. This review revealed the feasibility of predicting 3D shapes using ML clinically, which could play an important role for clinician-patient visualization and communication. However, all studies were early phase and there were inconsistent language and reporting. Future work could develop guidelines for publication and promote open sharing of source code.

**Supplementary Information:**

The online version contains supplementary material available at 10.1186/s12859-022-04979-2.

## Background

3D shapes of the human body are digital representations of physiological and pathological anatomical structures, acting as a bridge between virtual and physical worlds. There are three primary 3D shape file types, polygon mesh, point cloud, and 3D voxelised object, of which a mesh is the most common form [1, 2]. As virtual representations of the human body, 3D shapes enable non-invasive exploration of its components and potential automation of clinical solutions to design tissue scaffolds and medical devices [3].

3D surface scanning is the most common method of capturing external body morphologies, while medical images such as computed tomography (CT) and magnetic resonance imaging (MRI) are used to explore internal 3D body shapes. 3D surface scanned shapes can be less costly and more accessible than medical imaging and arguably more informative for representing the body's external appearance. Viewing 3D shapes of the body, either from surface scans or rendered from medical imaging, enables a more intuitive understanding of the relationship between anatomical features [4]. Machine learning (ML) is a field of artificial intelligence that designs algorithms for teaching machines to achieve tasks such as pattern detection, thus building an autonomous learning model [5]. ML is regularly applied to 2D images, and real-life 3D objects (such as vehicles and furniture) for purposes of classification and reconstruction [1, 6]. 3D shapes have also been employed as initial input data of the neural network-based ML model which was developed as shape autoencoder and decoder for predicting body shape deformation [7]. However, 3D shape prediction in medical domain has not been clearly defined and the definition of 3D shape prediction can vary. Thereafter, the concept can be defined as follows: *3D shape prediction is to reconstruct 3D anatomical structures from estimated morphological changes (e.g., before and after a procedure). Here, the input to the algorithm is a representation of 3D shape.* In this case, workflow is referred as the overall process required to predict, while the ML model is the specific network architecture used. The term 3D shape includes 3D volume, 3D mesh, 3D point cloud and other 3D representations, and the before-after (pre-post) operation cohorts can act as paired data.

Most work to date has focused on 2D data obtained from MRI or CT images and relates to object detection, segmentation, and disease classification [8, 9]. The application of ML driven 3D shape prediction in a clinical setting is currently lacking and an area ripe for investigation. As a relatively new area that sits at the intersection between computer science and clinical research, the current state of the art for 3D body shape prediction is unknown. Therefore, the aim of this systematic review was to identify and appraise ML methods for predicting 3D shapes focusing on clinical applications, prediction workflows, and prediction performance.

## Results

### Summary of the included studies

**Fig. 1:**
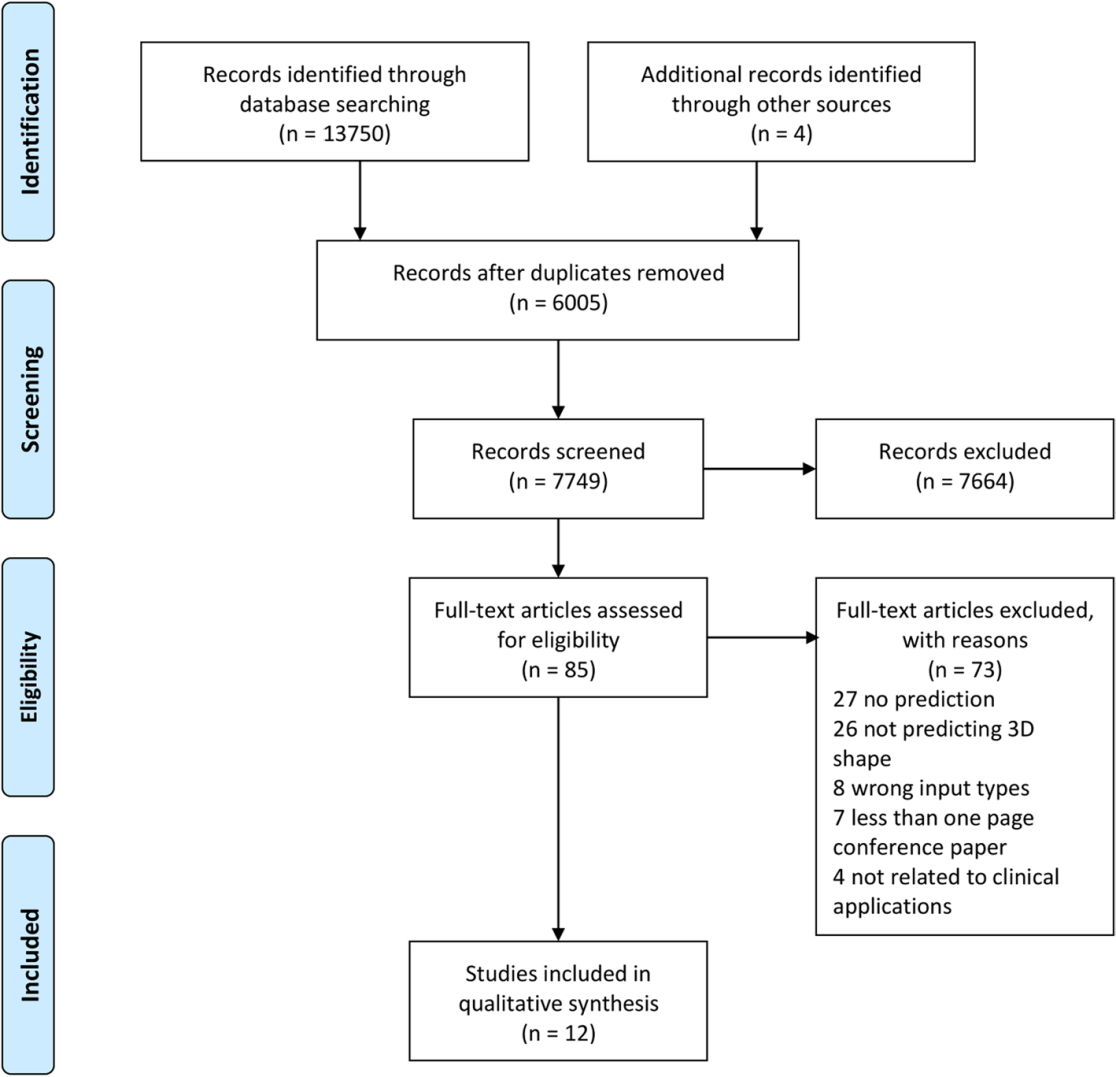
PRISMA flowchart. The inclusion and exclusion criteria are applied to the abstract-title screening and full-text screening process. Studies from citation searching were also identified and went through screening process

A total of 13,754 articles were identified, leaving 7749 after removing duplicates (Fig. 1). Following abstract and title screening, 7664 articles were removed, leaving 85 articles for full-text screening.12 articles met the inclusion criteria [10–21] including one identified through hand searching [14]. All 12 articles were published between 2015 to 2022. Six studies were identified as unspecific cohort studies [10, 11, 15, 18, 19, 21], five were retrospective cohort studies [12, 14, 16, 17, 20], and one was a case study [13]. 10 studies described approval from a relevant human ethics committee, however this was not mentioned in two articles [10, 15]. These studies predicted 3D shapes of a wide range of body regions, including the face [11, 14, 18–20], brain [10, 15], head [16, 21], forearm [12], aorta [13] and breast [17]. Sample sizes ranged from n = 7 to 209. Datasets with less than 50 samples occurred in 50% of studies, and none of the studies conducted a formal sample size calculation. The prediction models were developed with regression (67%), artificial neural networks (25%), and principal component analysis (8%). All methods were designed for development purposes without any external validation. The programming language was mentioned by 58% of the included papers. Python [14, 18, 20], C++ [11, 16, 19], and MATLAB [11, 18, 20, 21] were three languages used for building and running programs, with MATLAB the most prevalent. However, none of the articles published source code which would bring difficulties to open science and method replication.

### Clinical applications

3D shape prediction was used to assist with outcome visualization [10, 11, 15, 16, 18, 19], customized surgical planning [11, 14, 18–20], communication [12, 14, 18], diagnosis [10, 15], decision-making processes [13, 16] and implant generation [21]. We found most studies were exploring the use of 3D shape prediction as a tool for surgical planning and visualizing the aesthetic outcome. Use cases included simulating facial or breast changes after a procedure, predicting post-operated bone shape following orthopedic surgeries, soft tissue changes following denture implantation and implant design for cranioplasty.

All studies aimed to predict morphologies of different body regions from 3D shapes which were paired before and after an intervention or linked along timepoints. Six studies used 3D scanning to capture their shapes [11, 14, 17–20], of which two studies specified 3D scanning protocols such as head position and facial expression [18, 19]. Seven used medical imaging (MRI [10, 15] and CT [12, 13, 16, 18, 21]), while one study utilized both 3D shapes from 3D scanning and cone-beam CT scans as the inputs [18].

### Prediction workflow

Based on the studies retrieved, a common workflow for 3D shape prediction was identified: data pre-processing, 3D shape predictive model development (learning and predicting phases), and performance testing. Specifically, we divided the predictive model into two phases: relationships of morphological changes were derived in learning phase, while the prediction phase can be described as automatic generating new 3D shapes based on the learnt relationships. More details can be found in Table 1.

**Table 1:** General information of the included articles

	Body region	Clinical application	Machine learning categories	Workflow summary	Model performance	Comments
Cheng, 2015 [11]	Lower third of the face	Predict facial deformation of a patient after denture prosthesis. (outcome visualization, surgical planning)	Reinforcement learning	Register pre- and post-operative meshes Construct templates with feature points Predict changes with a neural network Simulate entire deformation	Error: − 2.021 to 2.021 mm, 95% of lower third face: < 2 mm Training/testing time: less than 10 s	Pipeline of prediction figure provided Two steps of prediction (coordinates of feature points, then the remaining area)
Do, 2019 [13]	Abdominal aorta	predict abdominal aortic aneurysm growth. (decision support)	Supervised learning	Reconstruct 3D shapes Reference registration Generate the IS field Build dynamic IS model Predict fields and uncertainty Generate AAA shape from the predicted field	Error: mean: 10.30 (3.62) mm	Method summarized with figures
Knoops, 2019 [14]	Face	Establish a large-scale clinical 3D morphable model. (Decision support, surgical planning, communication support)	Supervised learning	Scan patient face data Construct 3DMM Analyze characteristics of 3DMM Reduce high-dimensional manifold Classify face shapes into patient or non-patient group Predict 3D face shapes	Error: LARS: 1.1 ± 0·3 mm, RR: 1.1 ± 0·3 mm, LASSO: 1.3 ± 0.3 mm and LR: 3.0 ± 1.2 mm	The main purpose of the paper was not prediction Revealed the clinical potential of a large-scale clinical 3DMM using 3D scans (non-ionizing) and machine learning
Nguyen, 2020 [16]	head	Predict skull shape from a given head surface. (Outcome visualization, computer vision system aid for facial rehabilitation)	Supervised learning	Segment head and skull Reconstruct 3D meshes Register head and skull mesh in pairs Train PLSR model to obtain relationships Predict skull from new 3D head shape Deform the predicted 3D skull again based on generic skull mesh	Mean Hausdorff distance: 2.09 ± 0.15 to 2.64 ± 0.26 mm training/testing time: 9 min 4 s ± 10 s	Method summarized with figures Two steps of prediction (coast forecasting, then sophisticated reconstruction) No large sample size required to work well for PLSR Predictors are large but affect PLSR less than PCA
Oura, 2017 [12]	Radius and Ulnas	Predict whole bone shape from the partial shape. (Cost and radiation exposure reduction)	Supervised learning	Segment and generate 3D forearm shapes Identify landmarks, shape registration and 3D shape cutting Learn the relationship between whole and partial bone shapes Predict new whole shape from new partial bone shape	Error: 0.71–1.03 mm MATE and MARE: 0.48–1.76 mm and 0.99 -6.88 degrees	Method summarized with figures The model and workflow were not described in much detail
Rekik, 2016 [15]	Infant cortical shape	Build prediction model for longitudinally developing cortical surfaces in infants. (Diagnosis support, outcome visualization)	Supervised learning*	Estimate cortical surface growth Register baseline surfaces onto a common space Estimate temporal evolution for each baseline shape. A mean atlas is built for each timepoint Predict shapes by local shape morphing and the learnt features Deform surface by moving cloud points to the nearest neighbor	Mean surface error distance (mm): Left: 3-month:0.740 ± 0·727; 6-month:0.981 ± 0.949; 9-month:1.059 ± 1·007; 12-month:1.080 ± 1.039 Right: 3-month:0.756 ± 0·739; 6-month:1.037 ± 0.976; 9-month:1.068 ± 1·003; 12-month:1.115 ± 1.050	Prediction algorithm was specified No request of point-point surface correspondence Slight changed to their energy functionals and algorithms from 2015
Rekik, 2015 [10]	Infant cortical shape	Predict dynamic evolution of infant cortical shape. (Diagnosis support, outcome visualization)	Supervised learning*	Align and segment MR image Reconstruct 3D cortical shape. Convert surface shapes into current Build regression model based on the current metrics Register baseline shapes to a common space Estimate the temporal evolution trajectory, build mean atlases Deform surface based on existing clouds using two closeness metrics	Average distance errors: 3-month: 0.811 mm; 6-month: 0.953 mm; 9-month: 1.011 mm Average surface area difference (%) across all ROIs: 3-month: 7.8%; 6-month: 12.9%; 9-month: 15.4%	Prediction algorithm was specified Method summarized with figures Customized mathematical formulation for the experiments
Sampathkumar, 2020 [17]	Anterior torso from sternal notch to umbilical notch	Generate an estimation of the post-operative breast shape for cosmetic and reconstructive surgery. (Communication support)	Supervised learning	Conduct 1,320 SPHARM coefficients of pre- and post-op shapes Obtain transformation vectors Learn the relationship of transformation vectors Predict post-op shape based on the forecasted transformation vector and pre-op shape	RMSD (pre, post) = 17.24(5.57) RMSD (pre, predict) = 13.36(2.58) RMSD (post, predict) = 19.68(4.74)	Excellent with testing on training dataset, awful result with test data It was not feasible to forecast a single generalized change for all surgery types
ter Horst, 2021 [18]	Closed jaw	3D virtual soft-tissue simulation after mandibular advancement Surgery. (Surgical planning, outcome visualization, communication support)	Unsupervised Learning	Register pre- and post-op meshes (CPD). Get the mandibular displacement based on a reference mesh Predict the soft-tissue displacement Apply displacements to the pre-op vertices	Lower face: MAE: 1.0 ± 0.6 mm. RMSE: 1.2 ± 0.6 mm Lower lip: MAE: 1.1 ± 0.9 mm Chin region: MAE: 1.4 ± 0.9 mm	Neural network architecture was clearly illustrated with a figure. They compared the DL results not only with ground truth, also MTM shape
Tanikawa, 2021 [20]	Face	Predict 3D facial shape after orthognathic surgery and orthodontic treatment. (Surgical planning)	Unsupervised learning	3D scan faces Identify 18 landmarks and standardize with a common coordinate system Perform GMM to fit meshes Train model with pre-shape (6017 points), values of cephalometric landmarks, and changes of cephalometric landmarks Predict shape changes for new patients Sum pre-shape and predicted changes to form predicted post-shape	Result: average error (S):0.94 mm, average error (E):0.69 mm Abstract: error (S): 0.89 (0.30) mm, error (E): 0.69 (0.18) mm^†^	Neural network architecture was clearly illustrated with a figure Accuracy was reported differently between result and abstract sections
Wu, 2022 [21]	Head	Generate implant design for cranioplasty	Supervised learning	Derive 3D shapes from CT image Register shapes, augment and down sampling the data Train supervised learning model with flawed and intact 3D skull shape Predict intact shape from new flawed one^‡^	Only assessed by generated implant^§^ Volumetric error rate: Cylinder: 8.11%; Irregular cylinder: 8.04% Ellipsoid: 6.60%; Irregular ellipsoid: 7.17%	Neural network architecture was clearly illustrated with a figure
Yuan, 2017 [19]	Lower third of the face	Predict aesthetic reconstruction effects in edentulous patients. (Surgical planning, outcome visualization)	Unsupervised learning	Register edentulous and dentate meshes on the same coordinate system Construct feature template Construct soft tissue deformation Simulate entire deformation	Error (mm): Range: 1.090–0.480, mean: 0.769 ± 0.205 Statistically significant differences between participants Total run time: < 10 s	Contents were reported differently between method (PCA) and other sections (BP) No details of PCA prediction

IS, implicit surface; 3DMM, 3D morphological model; LR, linear regression; RR, ridge regression; LASR, least-angle regression; LASSO, least absolute shrinkage and selection operator regression; PLSR, partial least squares regression; PCA, principal component analysis; SPHARM, Fourier spherical harmonics; MATE, mean absolute translational errors; MARE, mean absolute rotational errors; ROIs, range of interests; P1, pre-op shape; P2, post-op shape; E, predicted shape; CPD, coherent point drift method; MAE, mean absolute error; RMSE, root mean square error; RMSD, root mean squared distance; DL, deep learning; MTM, mass tensor modelling; BP, back propagation; GMM, landmark-based geometric morphometric method analysis

*Some elements are semi-supervised. For example, a growth model was used while learning the dynamic cortical surface growth with missing data

^**†**^This paper had conflicting accuracies from result and abstract section. They also estimated two different interventions together: orthognathic surgery (S) and orthodontic treatment (E)

^**‡**^Further steps for generating implant shapes were eliminated since we only focused on 3D shape prediction

^**§**^This paper was main doing cranial implant design, 3D shape prediction was just a part of the workflow, therefore, they only assessed error of designed implant shape, no accuracy evaluated for skull shape which was predicted by the V-net

Data pre-processing for studies using medical imaging (MRI or CT) as input involved image segmentation, 3D shape registration and reconstruction using commercial (e.g., Materialise Mimics) or opensource software (e.g., Free3D and 3D slicer). Six articles highlighted 3D shapes reconstruction from medical images as one step of data pre-processing [10, 12, 13, 15, 16, 21], which were then used as inputs for the learning model. The iterative closest point method was the most common tool for 3D shape registration [11, 12, 19], while one employed coherent point drift algorithm [18]. A template shape with landmarks was employed as the reference for registration in two studies [11, 13].

The features used for prediction, ‘predictors’, had different types and numbers across 12 studies. Five studies introduced feature points/vertices with customized numbers and definitions [11, 16, 18–20], while two used shape or volume descriptors such as Fourier Spherical Harmonics coefficients [17]. Rekik *et al.* employed current and varifold metrics as predictors derived from customized mathematical equations [13, 18], and Do *et al.* developed the implicit surface that could define an object in space by mapping coordinates of points onto a scalar value [13]. 3D shapes of the radius and ulna, defined and cut according to identified anatomical landmarks, were employed as the predictor by Oura [12], while Knoops *et al.* did not specify the use of any form of predictors in their study [14]. Wu *et al.* mentioned 3D encoder-predictor network, whereas no predictor was clearly defined [21]. In addition, one study considered BMI as a confounding factor for 3D breast shape prediction [17].

A variety of ML algorithms were established across the 12 articles for learning phase of predictive model development. In total 14 ML algorithms were used, of which one was reinforcement learning [11], three was unsupervised learning [18–20], and all others were supervised learning [10, 12–17, 20, 21]. The ML model was reported inconsistently in one article, where principal component analysis was described in the methods while the rest of the article referred to back propagation network [19]. Knoops *et al.* tested four algorithms in their study: linear regression, ridge regression, least-angle regression, and least absolute shrinkage and selection operator regression [14]. Sampathkumar *et al.* investigated least square regression and random forest regression [17]. Partial least square regression was used by two different studies [12, 16]. Two articles from the same authors used a similar 4D (spatiotemporal) surface regression method, with the key differences in the mathematical surface representation: current (based on Faraday's law from Physics) and varifolds [10, 15]. Neural networks, including back-propagation neural network and autoencoder-inspired neural network, were used by Cheng and ter Horst [11, 18]. Tanikawa et al. mentioned regression using customized deep neural network [20], while a V-net was used by Wu *et al* [21]. Principal components analysis and spatiotemporal Gaussian regression with Expectation-Maximization Kalman filter were identified in another two studies [13, 19]. Algorithm architecture and features such as the number of hidden layers of neural networks and parameter settings can be seen in Table 2. The relationships learned by the regression models allow them to predict new 3D shapes external from the training dataset. In addition, Knoops et al. mentioned overfitting occurred in linear regression as a limitation but without a solution [14], however Tanikawa *et al*. mentioned the use of Dropout layers for reducing the chance of overfitting in neural network [20]. No other papers mentioned prevention of overfitting.

**Table 2:** Details of machine learning model development of the included studies

	**Sample size**	**Training/ testing size**	**Predictors**	**Algorithm name**	**Algorithm description**
Cheng, 2015 [11]	48	43/5	29 feature points on the lower third face	Back propagation neural network	An input layer with 29 nodes, a hidden layer with 20 nodes, and an output layer with 29 nodes
Do, 2019 [13]	7 age range: 54–73 (100% male)	NA*	IS field	Spatiotemporal Gaussian Process and EM filter (Kalman)	Gaussian process constructs the IS An EM filter (Kalman) estimates parameter of temporal evolution of the IS field
Knoops, 2019 [14]	N = 151, mean age: 18·4 (2.4), age range: 14–28 (56% female)	Not clear^†^	Not mentioned	LR RR LARS LASSO	RR and LASSO: the alpha was set to 0·5 and 0·1 LARS, the number of non-zero coefficient was set to 1 All the other parameters were kept the default values All regression methods but LR penalized the weight of the components with a regulizer
Nguyen, 2020 [16]	209 age range: 34–88 (23% female)	146/63	Head feature points Feature distances Volumes	PLSR	Get the predictor and response variables (thickness and matrix Train the model coefficient matrix B Predict response variables based on given predictor variables using B
Oura, 2017 [12]	100 mean age: 45.5, age range: 16–85 (47% female)	80/20	Proximal 60%, Distal 60%, Distal 30% and proximal 30%	PLSR	The correlation A was learnt from the training dataset (Y _(whole shape)_ = AX _(partial shape)_)
Rekik, 2016 [15]	12	11/1	Varifold metric	Varifold-based geodesic shape regression	Longitudinal varifold-based shape regression. Fitting the deforming baseline shape into a set of target shapes by minimizing the energy functional The regression was used to link all subjects' longitudinal shapes in space and time A dynamic cloud was generated to model the temporal evolution trajectories of the baseline geometric shapes Virtual shapes were constructed for prediction. Searches of the local topography were used to estimate the geodesic evolution of the shape for a new subject
Rekik, 2015 [10]	17	14/3	Current metric	Spatiotemporal current-based surface regression	A 4D surface growth model is trained to learn the deformation of the baseline shape in consecutive timepoints by the diffeomorphic mapping An external momentum of the change locally acts on baseline shape's Dirac delta currents, which deforms to consecutive shapes. The momenta define the surface deformation process by conjugate gradient descent algorithm minimizing the energy functional Virtual shapes were constructed for prediction. Searches of the local topography were used to estimate the geodesic evolution of the shape for a new subject
Sampathkumar, 2020 [17]	33 pre-op age range: 24–68, post-op age range: 27–71	21/41	SPHARM 1320 (440*3) coefficients	Least square regression Random Forest regression	A random forest regression was trained to learn the non-linear relationship between the transformation vectors SPHARM coefficients of the pre-op breast with the transformation vector predicts post-op shape using least squares optimization
ter Horst, 2021 [18]	133 mean age: 29.5, age range: 14–65 (53% female)	119/14	3129 vertices 5 nodes for mandibular displacements	Deep learning: autoencoder neural network	Six dense layers in full network Each dense block had a Leaky ReLU activation function Batch normalization momentum: 0·5 Dropout rate: 0·5
Tanikawa, 2021 [20]	Surgery group: 72; orthodontic group: 65	Data separated into 11 sets: 10 training, 1 testing	6017 points on pre-treatment shape, with values of 27 cephalometric landmarks, and changed values of 16 cephalometric landmarks	Deep learning: customized neural network	Two dense layers with ReLU activation function One dropout layer (0·3) Adam optimizer for optimization MSE for loss function
Wu, 2022 [21]	73	7154(10% for testing)	Flawed 3D cranial model with a volumetric resolution of 112*112*40	High dimensional autoencoder augmented with skip connections (V-Net)	Twelve 3D convolutional layers: four 3D expansion layers, three max-pooling layers, three up-sampling layers, augmented with eight skip connections 8269 trainable parameters
Yuan, 2017 [19]	10 mean age: 73.2 (4.3), age range: 68–80 (50% female)	Not mentioned	29 feature points on the lower third face	PCA^‡^	Reduce dimensionality on a multi-dimensional variable system PCA is an extraction method based on the minimum mean square error. The completed PCA model is also the facial elastic deformation prediction model, which can predict the elastic deformation of the edentulous model feature template

NA, not applicable, IS, implicit surface, EM, expectation maximization, LR, linear regression, RR, ridge regression, LASR, least-angle regression, LASSO, least absolute shrinkage and selection operator regression, PLSR, partial least squares regression, PCA, principal component analysis, SPHARM, Fourier spherical harmonics, LSFM, Large-scale facial model

*In this paper, each of the 7 patients had multiple CT images (e.g., some with 4). A per patient training scheme was used and the last was used as the ground truth and the rest (e.g., first 3 out of 4) as training. There was no test set because each model was personalized for a patient

^†^For shape prediction, the paper talks about three 3D morphological model: a global model, a bespoke pre-operative model, and a bespoke post-operative model. The global model (n = 4216) comprised all patient scans as well as healthy volunteer scans from the same age range. The bespoke pre-operative (n = 119) and post-operative (n = 127) models were made exclusively with patient scans

^‡^Method was documented inconsistently in abstract, results, discussion, and conclusion

Regarding to the predicting phase, two articles distinguished it from the learning phase, in which specific algorithms were provided [10, 15]. Shape post-processing were also found after prediction using different computing methods. Two articles highlighted the application of the Laplacian deformation technique for generating predicted shape [11, 19]. Moreover, one study had proposed a post-prediction process of generating a 3D skull with high resolution from a coarse deformed skull via three steps: registration, initial deformation, and refinement deformation [16]. Two study used a post-prediction process to generate post-operative shape by applying predicted displacement onto the pre-operative shape using MATLAB, however no further details were provided [18, 20].

No studies reported any differences between the training, testing, and validation datasets for each prediction model in inclusion criteria, model outcome, or predictors. Moreover, all studies had built prediction models for only early-stage development that used internal validation alone. Three out of 12 papers used leave-one-out validation [12, 14, 15], whereas ten-fold cross-validation was employed by Nguyen [16], and 11-fold by Tanikawa [20]. Others indicated customized methods, for instance ter Horst *et al.* [18] validated their deep-learning based prediction against mass-tensor-model prediction. However, no validation method were mentioned by Wu [21].

### Prediction performance

Different methods were employed to evaluate performance of the prediction among included articles, which limited the ability to compare between studies. Seven articles displayed results with both figures of 3D shapes and heatmaps to indicate the predicted shapes and accuracy [10, 11, 14–16, 18, 20]. Three articles showed 3D shapes only [13, 17, 21], and two reported neither figures nor heatmaps [12, 19]. All studies reported acceptable prediction error ranging from 0.69 to 19.68 mm, although one had conflicting accuracy presented between result (average error 0.94 mm for surgery group) and abstract (average error 0.89 mm for surgery group) [20], and another one only highlighted accuracy of the designed cranial implant shape rather than predicted skull shape [21] (see Table 1). The prediction accuracy was assessed by comparing the predicted 3D anatomical structures and ground truth using either Hausdorff distance or Euclidean distance. Due to the differences of investigated body regions and methods for calculating the accuracy, we were unable to conduct a meta-analysis. In addition, four articles highlighted training/testing duration, two of which reported less than 10 s [11, 19], one was 9 min 4 ± 10 s [16], whilst Wu specified 58.4 h. for training and 8.6 s for testing [21]. No studies reported any calibration measures of the performance.

### Quality assessment

All papers were rated as having an overall high risk of bias using the Quality In Prognosis Studies (QUIPS). The rating of each domain can be seen in Table 3, where color intensity distinguishes high, moderate, low risk of bias, and grey indicates a domain that was considered irrelevant. Unclear descriptions and limited information led to low scores in quality assessment. A few papers failed to report participant information, inclusion, and exclusion criteria, which led to a high risk of bias in the domain of study participants. The main reason for the moderate rating for prediction factor and outcome measurement domains was the lack of a reliability test for measurement methods. Four out of 12 studies mentioned their models for statistical analysis [12, 18–20], whereas three rated high since no model for statistical analysis was mentioned and the predicted 3D shapes were reported with bias [10, 17, 21].

**Table 3:**
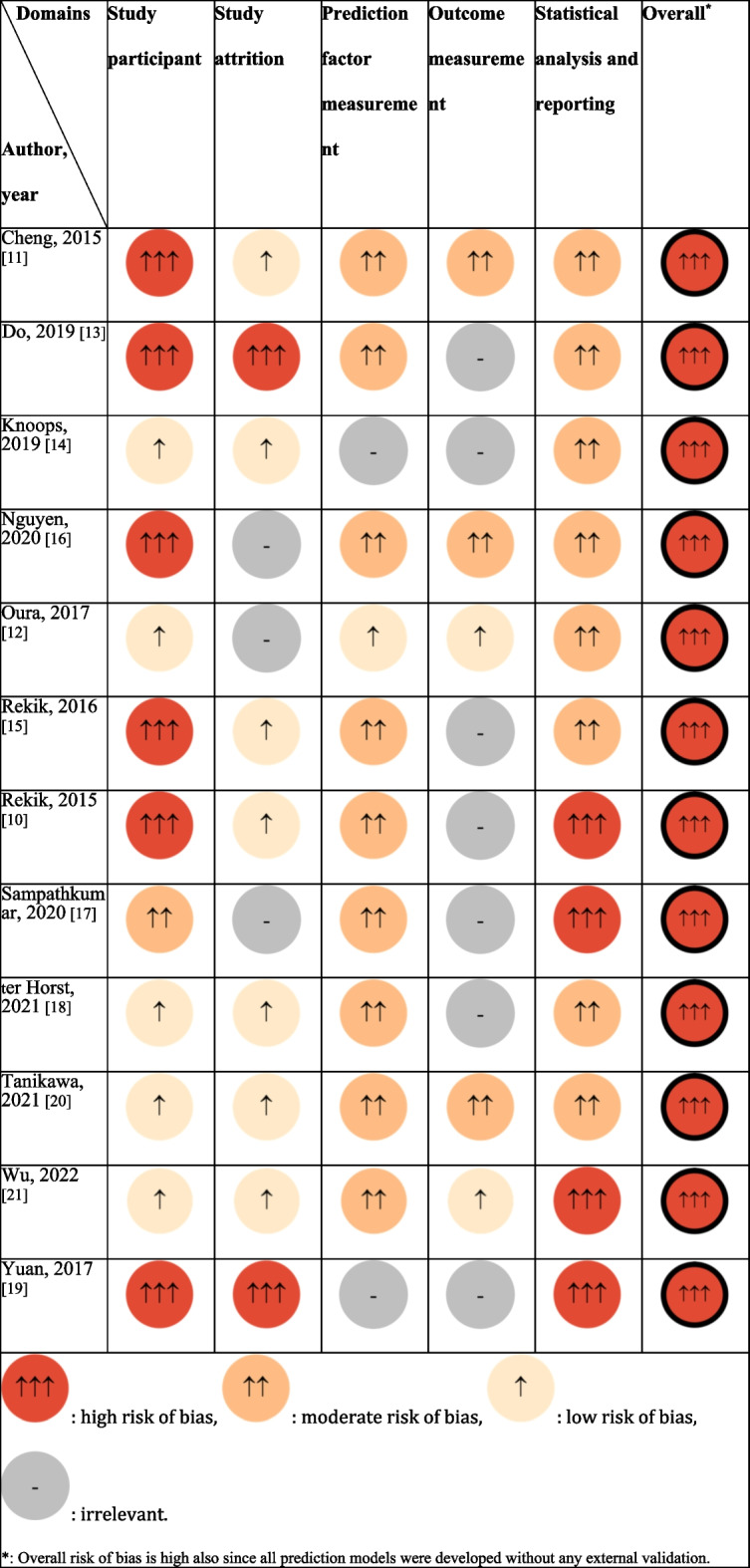
Risk of bias as assessed by Quality In Prognosis Studies (QUIPS)

## Discussion

This systematic review found 3D shape prediction using ML has potential for post-operational anatomy visualization, however all studies were in preliminary phases. All included studies focused on the upper body, with more than half predicting shapes from the head area. Neural network-based methods become more popular in studies since 2021 compared to other methods, such as regression. Although all studies claimed promising results, some drawbacks can be identified such as the inadequate information for model validation with accuracy and unreliable reporting of methods. Critically, none of the included studies shared source codes, which limits reproducibility. Other deficiencies such as undefined standard of reporting, terminology consistency and evaluation were also recognized and should be discussed.

Prediction of 2D images has progressed dramatically and has many proposed applications in diagnostics, prognostics, and clinical decision aid tools [8, 9]. CT and MRI modalities are also commonly used to train convolutional neural networks as 2D data sets [22]. However, there may be additional benefits for ML driven predictions of 3D datasets in clinical settings. The ability to predict 3D shapes enables intuitive visualization of outcomes, which is useful for research focused on anatomical shapes such as the studies on the skull, radius and cortex included in this systematic review [10, 12, 16]. In some clinical scenarios, an external 3D surface scan of the body is preferred over other imaging modalities for understanding changes in the body’s appearance, for example, the ability to visualize post-operative facial soft tissue deformation via 3D shape prediction [11, 18, 19]. Reducing the reliance on CT scanning for external features by using 3D surface scanning also has the potential to reduce radiation exposure for patients [11, 16]. Furthermore, some applications can only be conducted using 3D surface scanning, such as those require soft tissues in a particular conformation that are difficult or impossible to capture in conventional CT or MRI. These may include standing or weight-bearing views, which are important in orthotics and prosthetics, such as for prosthetic socket design [23]. The ability to apply ML and prediction algorithms to 3D shapes is essential for these applications. However, implementing ML approaches for 3D shape prediction into a clinical setting may be challenging. Obtaining regulatory approval will prove difficult and finding a leading indication will be important. Other obstacles will likely include a lack of infrastructure and resources to support large databases for validation and computing power, data security and privacy, difficulty integrating into existing workflows, and overcoming clinicians’ distrust [24].

**Fig. 2:**
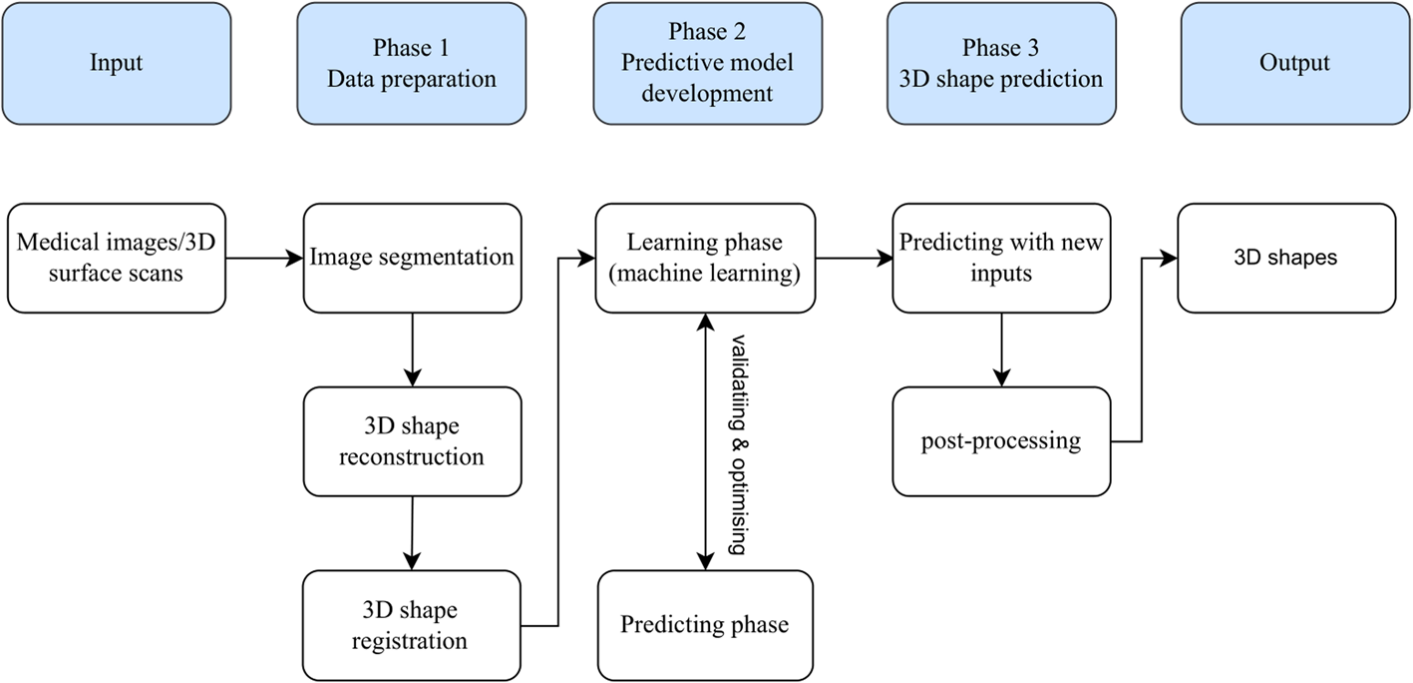
General workflow of 3D shape prediction summarized into three phases. Phase one: Data preparation including segmentation, reconstruction, and registration. Phase two: predictive model development including two phases: learning and predicting. Phase three: 3D shape prediction based on the predictive model developed and optimized in phase two

The methodologies of included studies shared common approaches and deficiencies. We summarized the 3D shape prediction workflow from the included studies into three phases: data preparation, predictive model development, and 3D shape prediction (Fig. 2).

Data pre-processing procedures were often scant on details such as re-mesh methods, registration parameters, and 3D shape properties in terms of the number of vertices and their simplicity methods. A possible reason could be that most studies used software to process the 3D shapes, and the unknown algorithms applied behind a "click and go" approach means the pre-processing occurs inside a black box. Although some studies have defined the number of vertices on their 3D shapes, there was usually no evidence to justify their selection and why it was appropriate for their studies. This review revealed a broad range of ML models used for 3D shape prediction, of which the most popular were neural networks followed by partial least squares regression, while papers published since 2021 all used deep learning neural networks. The advantages of artificial neural networks are that they can fit complex nonlinear models and deal with high-dimensional data [25], which is suitable for 3D shape prediction. However, the architecture of neural networks can be complex to design, usually requires a large database for training, and can be subject to overfitting [25]. Other regression algorithms, such as linear regression, are simpler to understand and implement, however they might result in low accuracy and overfitting. Further, learning and predicting phases were usually not clearly defined and reported in the study. For example, some studies partially reported pre-set parameters while others did not report any information on parameter settings [11, 17], which can lead to reproducibility issues. The satisfactory results of prediction accuracy could also be concerning as only one study had mentioned clinically acceptable accuracy in the article [18]. Unfortunately, study quality did not improve over time, thus there is an urgent need for establishing standards for terminology and reporting guidelines.

The inconsistent language used around 3D shape prediction was one of the biggest challenges for searching and screening articles. Some studies mentioned prediction in the title or abstract, despite the content relating to other topics such as pure shape generation, and vice-versa. Others used alternative keywords such as transformation and virtual simulation when these studies related to prediction. Moreover, the inconsistent usage of terminologies for model development was identified such as the word ‘validation’ and ‘testing’ for performance evaluation [12, 17]. The mixed understanding of the two words has introduced significant inconsistencies across the studies. The field would benefit from the introduction of best practice guidelines specific to the 3D domain, as has been done for biomedical 2D image analysis with recommended terminologies and reporting guidelines [26].

We conducted a systematic review, which is a broadly accepted approach in medical research, to identify and summarize studies of 3D shape prediction for clinical uses. The systematic review approach overviews the current existing knowledge on the given topic and identifies inconsistencies, gaps, and future directions. It is conducted following an explicit protocol using PRISMA, which assures a comprehensive search with clear inclusion and exclusion criteria. Well-established standards are also used to extracting information, reporting findings, and assessing study quality. For these reasons, the systematic review is a strong method of surveying current evidence and to guide future research of a topic. However, our goal in this study was to identify any fields of medicine predicting 3D shapes, as such we did not use specific body region terms in our search. With the growth of 3D body shape prediction, future systematic reviews may benefit from specialty-oriented searches. This systematic review also used a language limitation, and studies conducted in languages other than English would likely be missed. Regarding the scope of this review, suitable methods applied in non-medical fields such as gaming and computer vision may have also been excluded. For example, in this systematic review studies in forensics were excluded since they were deemed to be out of the scope of this review [27–29]. Moreover, QUIPS checklist is not specifically developed for ML applications, however, it has been employed by other studies related to ML prediction models for clinical applications [30, 31]. Other checklists has also been utilized to evaluate quality for prognostic studies within the same domains as QUIPS [32]. However, they do not include assessment about ML related biases. For example, an algorithmic bias could occur where a study may apply popular algorithms and adopt prevalent algorithmic design choices that are suited for certain dataset. A bias could also occur during the model evaluation process since no appropriate benchmarks for evaluating the application were well defined.

There has been great progress in 3D shape prediction since the first study in our review was published in 2015. The most promising applications of 3D shape prediction seem to focus on visualizing and communicating the physical changes following surgery. However, several challenges need to be overcome before the field advances further. Here, we list some areas of focus and future research that could potentially improve study quality and support clinical translation.

1. *Improved dataset size.* A high-performance ML model requires a considerable amount of reliable training data [9]. Therefore, larger datasets or extensive published databases would benefit studies creating exploratory prediction models. Similar to adjacent fields, a community for sharing data for use in training and evaluation, such as The International Skin Imaging Collaboration [33], could be established.
2. *More formalized evaluation protocol with separate training, validation, test phases/datasets.* It is crucial that training, validation, and testing datasets should never overlap and must be clearly defined before developing models. Training datasets are adapted for regression model to learn relationships; validation datasets are used for evaluation during development to tune model hyperparameters and to optimize the model, while testing datasets are for final estimating the developed model.
3. *Development of a set of standard guidelines for comparability.* A guideline for publishing studies on 3D shape prediction could be established to improve the structure, design, and reporting of studies, which is important if the goal is clinical implementation. Specifically, methods of evaluating prediction accuracy and clinically acceptable accuracy should be assessed and discussed. An AI extension of the Transparent Reporting of a multivariable prediction model of Individual Prognosis Or Diagnosis (TRIPOD) is currently under development, and we recommend a further 3D shape specific version [34]. Some 3D shape specific items could include predictors, computing hardware, software for AI model development, and a flowchart for demonstrating the entire workflow.
4. *Standardization of nomenclature.* Unifying language through agreed definitions would enable greater consistency. For example, the use of ‘prediction’ as the general keyword for many articles that describe the forecasting of 3D shapes, while other terms were also employed such as ‘reconstruction’ or ‘simulation’.
5. *Sharing Source code.* Sharing of the source code publicly, such as an appendix or on an online repository, will promote open science, replication of the results, and support research collaboration.

## Conclusion

ML has been used in 2D medical imaging analysis since the early 1990s and rapidly expanded since 2015, but it is only recently that 3D shape prediction has been applied to clinical research. In this study, we found 12 publications that predicted six different regions of human body using 14 ML algorithms. Most of these studies had the goal of simulating surgical outcomes, all were early-stage research and were some ways from clinical implementation. However, these studies lacked consistent keywords and reporting structures. The nascent but evolving field of 3D shape prediction has great potential to improve medical scenarios that involve understanding shape changes before and after an intervention.

## Methods

### Search strategy and selection criteria

In this systematic review, we searched for studies that used ML to predict 3D shape for clinical applications. The search was conducted according to the Preferred Reporting Items for Systematic Reviews and Meta-Analysis (PRISMA) statement guideline. Four electronic databases, Medline, Embase, Scopus and Web of Science, were searched for studies published in English full text until 28th March 2022. Studies were identified with search terms including “three-dimensional imaging/”, “artificial neural network”, “machine learning” and “predictions”. The full search strategy is given in Additional file 1: Table S1. Manual searches were conducted on reference lists of the included articles to identify relevant publications.

We did not limit the target population, specific disease, or the category of the prediction model, however we had exclusion criteria for inputs, outputs and broad study areas. The inclusion criteria were predefined as: (1) inputs being two or more datasets for each participant, for instance, paired shapes (before and after) for one participant; (2) outputting a form of 3D shape; (3) paper reporting any 3D shape prediction model using at least one ML technique and its entire workflow; (4) study having clinical relevance aiming to improve medical conditions. Publications were excluded without English full texts or based on animal studies. Short conference abstracts (less than one page) and reviews (i.e., conference review) were excluded, as these do not contain enough detail for our review. This study is registered with PROSPERO, CRD42021263000.

The eligibility assessment, including title/abstract screening and full-text screening, was conducted using the Covidence (Covidence systematic review software, Veritas Health Innovation, Melbourne, Australia.) independently by two reviewers (JZW and JL). Duplicates were automatically and manually removed. Disagreements were first resolved by the two reviewers through discussion, then referred to a third reviewer (TLC or AK) if consensus was not achieved. Consensus was achieved for all included studies.

### Data analysis

Two reviewers, JZW (Bachelor of Engineering (chemical), Master of Professional Engineering (biomedical), PhD candidate (health science & machine learning)) and JL (Bachelor of Engineering) extracted data including basic information, sample population, method, outcomes, and clinical area using a predefined data extraction template on Covidence website. If the two reviewers did not agree on the inclusion of an article during screening or eligibility assessment, the article was referred to a third reviewer for further discussion until all reviewers agreed. The third reviewer was either TLC (Bachelor of Engineering (biomedical)/ Bachelor of Medical Science, PhD (medicine)) or AK (Bachelor of Engineering (Software Engineering)/ Bachelor of Arts, PhD (computer science)) depending on expertise.

The QUIPS tool was used to assess the risk of bias, which is originally designed to assess prognostic factor studies, however it can be used in other prognostic studies by removing or adjusting certain domains [35]. Reviewers (JZW, JL) independently evaluated five domains of QUIPS including study participation, study attrition, prediction factor measurement, outcome measurement, and statistical analysis and reporting, of which the study confounding domain was omitted (Additional file 2: Table S2). The overall risk of bias score for each study was assessed regarding to the rule adopted and modified from Grooten [36]: if all domains were rated as low risk, then the study was categorized having overall low risk of bias. A study with at least one domain rated high or more than two domains rated moderate was judged as overall high risk of bias. Other situations were categorized as overall moderate risk of bias. Moreover, if a prediction model was for development purpose only based on small database without any external validation, the study would be downgraded to high risk of bias even if it was listed as low risk of bias for all domains [37]. Meta-analysis was not appropriate for this review due to the differences in targeted body regions and various indicators for prediction accuracy.

## Supplementary Information

**Additional file 1.** Search strategy: key words and the methods used for literature search.

**Additional file 2.** The questions and strategies for assessing risk of bias.

## Data Availability

All papers are available on publisher websites. All data generated or analyzed during this study are included in this published article.
