# Supplementary material for: Clinical applications of machine learning in predicting 3D shapes of the human body: a systematic review
Source: BMC Bioinformatics. 2022 Oct 17;23:431. doi: 10.1186/s12859-022-04979-2 (PMC9575250; doi:10.1186/s12859-022-04979-2)
Supplement: Supplementary file 1 — Additional file 1. Search strategy: key words and the methods used for literature search. [file 12859_2022_4979_MOESM1_ESM.docx]

## Supplementary material

**Table S1. Search strategy**

| **Database** | **Search terms** |
| --- | --- |
| Medline | imaging, three-dimensional/  3d.tw  3 dimension*.tw  three dimension*.tw  Tomography/  Neural Networks, Computer/  exp Artificial Intelligence/  (supervised learning or Unsupervised learning or Reinforcement Learning or pattern recognition* or Pattern classification or back propagation or Naive Bayes or Random forest or Gaussian process or lasso* or elastic net* or extreme learning machines or case based reasoning or Long Short Term Memory or kernel* or genetic algorithm* or regression or principal component* or statistical shape*).mp  ((deep or convolutional or neural or bayesian) adj3 network*).mp  ((vector adj3 machine) or ((machine or deep or ensemble) adj3 learning)).mp  (nearest adj1 neighbo*).mp  ((classification or regression or probability) adj3 tree*).mp  ((auto* or virtual or change* or deform* or "elastic deform*" or outlier or surface or morpho* or shape* or geometr*) adj5 (predict* or variation or generati* or reconstruct* or forecast*)).tw. |
| Embase | three-dimensional imaging/  computer assisted tomography/  3d.tw  3 dimension*.tw  three dimension*.tw  artificial neural network/ or machine learning/ or autoencoder/ or back propagation neural network/ or cellular neural network/ or cohen grossberg neural network/ or complex valued neural network/ or deep neural network/ or discrete time neural network/ or feed forward neural network/ or fractional order neural network/ or functional link artificial neural network/ or memristive neural network/ or pulse coupled neural network/ or quaternion valued neural network/ or radial basis function neural network/ or reaction diffusion neural network/ or recurrent neural network/ or spiking neural network/  artificial intelligence/  (supervised learning or Unsupervised learning or Reinforcement Learning or pattern recognition* or Pattern classification or back propagation or Naive Bayes or Random forest or Gaussian process or lasso* or elastic net* or extreme learning machines or case based reasoning or Long Short Term Memory or kernel* or genetic algorithm* or regression or principal component* or statistical shape*).mp  ((deep or convolutional or neural or bayesian) adj3 network*).mp  ((vector adj3 machine) or ((machine or deep or ensemble) adj3 learning)).mp  (nearest adj1 neighbo*).mp  ((classification or regression or probability) adj3 tree*).mp  ((auto* or virtual or change* or deform* or "elastic deform*" or outlier or surface or morpho* or shape* or geometr*) adj5 (predict* or variation or generati* or reconstruct* or forecast*)).tw. |
| Scopus | 3d OR "3 dimension*" OR "three dimension*"  "supervised learning" or "Unsupervised machine learning" or "Reinforcement Learning" or "pattern recognition*" or "Pattern classification" or "back propagation" or "Naive Bayes" or "Random forest" or "Gaussian process" or lasso* or "elastic net*" or "extreme learning machines" or "case based reasoning" or "Long Short Term Memory" or "genetic algorithm*" or regression or "principal component*" or "statistical shape*" OR ((deep or convolutional or neural or bayesian) W/3 network*) OR (vector W/3 machine) or ((machine or deep or ensemble) W/3 learning) OR (nearest W/1 neighbo*) OR ((classification or regression or probability) W/3 tree*)  ((deep or convolutional or neural or bayesian) W/3 network*)  (vector W/3 machine) or ((machine or deep or ensemble) W/3 learning)  nearest W/1 neighbo*  ((classification or regression or probability) W/3 tree*  (auto* OR virtual OR change* OR deform* OR "elastic deform*" OR outlier OR morpho* OR shape* OR geometr*) W/5 (predict* OR variation OR generati* OR reconstruct* OR forecast*) |
| Web of Science | 3d OR "3 dimension*" OR "three dimension*" (Topic) and ( "supervised learning" OR "Unsupervised machine learning" OR "Reinforcement Learning" OR "pattern recognition*" OR "Pattern classification" OR "back propagation" OR "Naive Bayes" OR "Random forest" OR "Gaussian process" OR lasso* OR "elastic net*" OR "extreme learning machines" OR "case based reasoning" OR "Long Short Term Memory" OR "genetic algorithm*" OR regression OR "principal component*" OR "statistical shape*" OR ( ( deep OR convolutional OR neural OR bayesian ) NEAR/3 network* ) OR ( vector NEAR/3 machine ) OR ( ( machine OR deep OR ensemble ) NEAR/3 learning ) OR ( nearest NEAR/1 neighbo* ) OR ( ( classification OR regression OR probability ) NEAR/3 tree* ) ) (Topic) and ( auto* OR virtual OR change* OR deform* OR "elastic deform*" OR outlier OR morpho* OR shape* OR geometr* ) NEAR/5 ( predict* OR variation OR generati* OR reconstruct* OR forecast* ) |
