# Supplementary material for: Clinical applications of machine learning in predicting 3D shapes of the human body: a systematic review
Source: BMC Bioinformatics. 2022 Oct 17;23:431. doi: 10.1186/s12859-022-04979-2 (PMC9575250; doi:10.1186/s12859-022-04979-2)
Supplement: Supplementary file 2 — Additional file 2. The questions and strategies for assessing risk of bias. [file 12859_2022_4979_MOESM2_ESM.docx]

Table S2, Risk of bias assessment tool (QUIPS)

| **Biases** | **Issue to consider for judging overall rating of “risk of bias”** |
| --- | --- |
| **1. Study participant** | **Goal: to judge the risk of selection bias** |
| Source of target population | The study sample adequately represent the population of interest? i.e., both male, female participants |
| Inclusion and exclusion criteria | Adequate description of inclusion & exclusion? |
| Methods to identify population | Adequate description of period & recruitment location? |
| **2. Study attrition** | **Goal: to judge the risk of attrition bias** |
| Reasons of subjects lost to follow up | Reason for loss to follow-up are provided? |
| Information on those lost to follow up | Adequate description of participants loss to follow up? i.e., gender, age, disease-related info. |
| **3. Prediction factor (PF) measurement** | **Goal: to judge the risk of measurement bias related to how the PF was measured** |
| Definition of the PF | A clear definition and description of PF is provided? |
| Valid and reliable measurement of PF | Is there a reliability study mentioned in the paper? |
| Consistency of PF measurement | The method of measurement of PF is same for all participants? |
| **4. Outcome measurement** | **Goal: to judge the risk of bias related to the measurement of outcome** |
| Definition of the outcome | A clear definition of outcome is provided? |
| Valid and reliable measurement of outcome | Is there a reliability study mentioned in the paper? |
| Consistency of outcome measurement | The method of measurement of outcome is same for all participants? |
| **5. Statistical analysis and reporting** | **Goal: to judge the risk of bias related to the statistical analysis and presentation for results** |
| Model declaration | Provide reasons of choosing the statistical analysis model and description of the model? |
| Data presentation | Sufficient data presentation? i.e., 3D shapes and colourmap |
| Reporting of results | All variables (outcome and predictors described in method) are included in the result with words or numbers? |
